# Impact of imiglucerase supply constraint on the therapeutic management and course of disease in French patients with Gaucher disease type 1

**DOI:** 10.1186/s13023-015-0275-0

**Published:** 2015-05-13

**Authors:** Jérôme Stirnemann, Christian Rose, Christine Serratrice, Florence Dalbies, Olivier Lidove, Agathe Masseau, Yves-Marie Pers, Camille Baron, Nadia Belmatoug

**Affiliations:** Department of General Internal Medicine, Geneva University Hospital, Rue Gabrielle-Perret-Gentil 4, CH-1211 Genève 14, Switzerland; Department of Oncology and Hematology, Saint Vincent de Paul Hospital, Nord de France University, Lille, France; Department of Internal Medicine, Fondation Saint Joseph Hospital, Marseille, France; Department of hematology, Morvan Hospital, CHRU, Brest, France; Department of Internal Medicine, Croix Saint Simon Hospital, Paris, France; Department of Internal Medicine, CHU Nantes, Nantes, France; Clinical Immunology and Osteoarticular Diseases Therapeutic Unit, CHRU, Lapeyronie, Montpellier, France; Medical Department, Genzyme SAS, Saint Germain en Laye, France; Referral Center for Lysosomal Diseases and Department of Internal Medicine, CHU Paris Nord Val de Seine, Clichy, France

**Keywords:** Gaucher disease, Imiglucerase supply constraint, Enzyme replacement therapy

## Abstract

**Background:**

In 2009, a worldwide supply constraint of imiglucerase led to treatment modifications or interruptions for patients with Gaucher disease (GD) type 1. In France, joint treatment recommendations were issued to protect the most vulnerable patients. This observational study evaluated the impact of imiglucerase treatment modifications on the clinical and biological course of GD.

**Methods:**

Retrospective data on patients’ characteristics, treatment, clinical and biological parameters from 01 June 2009 to 31 October 2010 were collected during a single visit.

**Results:**

Ninety-nine GD1 patients, aged 7–84 years, were included (median age 47 years); 10 were children. Patients experienced a median of 4 different treatment modifications. Median change from pre-supply constraint dose (92 U/kg/4-weeks) was −69, −51, −29 and −60 U/kg/4-weeks at 3, 6, 9 and 12 months after first modification, respectively, with imiglucerase discontinuation reported for 70%, 47%, 29% and 55% of patients at these timepoints. Replacement with another ERT was reported for 35 patients. Results show a statistically significant decrease in hemoglobin (−0.8 g/L/month) and platelets (−5905.10^3^/mm^3^/month) and an increase in chitotriosidase (+537 nmol/mL/h/month) and angiotensin-converting enzyme (+4 IU/L/month) in the subgroup of 61 patients who discontinued treatment for at least 3 months; this magnitude of change was not seen in the subgroup (32 patients) treated with reduced imiglucerase for at least 3 consecutive months. GD-related events were spontaneously reported by the study investigators for 39% of the whole study population, including asthenia/fatigue (8%), bone infarction and bone pain (4% each), and hepatomegaly (3%). A Kaplan-Meier estimate of the probability for a patient to present a bone, hematological or visceral event during the constraint was 37% for patients who discontinued the treatment and 10% for patients treated with a reduced imiglucerase dose.

**Conclusion:**

The release of recommendations and individuals’ close follow-up allowed satisfactory management of patients during the imiglucerase supply constraint in France. This study suggests that during this period, lowering the dose of imiglucerase had less impact on the outcomes of patients than interrupting treatment. However, general effects (such as fatigue, bone pain) reported in some patients, emphasize the importance of maintaining appropriate individualized dosing.

## Background

Gaucher Disease (GD) is an inherited lysosomal storage disease, attributable to glucocerebrosidase enzyme deficiency; in rare cases, the absence of saposin C, an activator of glucocerebrosidase, leads to a Gaucher-like disorder [1]. GD is a rare autosomal recessive disease with an incidence between 1/50,000 and 1/100,000 in the general population, though it is more prevalent in the Ashkenazi Jew population [2]. GD is characterized by the presence of lipid-engorged macrophages whose nuclei are off-center (Gaucher cells). Abnormal reticuloendothelial cells mainly localize in the liver, spleen and bone marrow and their accumulation leads to the main signs of disease: hepatosplenomegaly associated with abdominal discomfort, thrombocytopenia and coagulopathy leading to a bleeding tendency, anemia associated with chronic fatigue, bone lesions that may become debilitating (e.g., chronic bone pain, bone crises, and/or bone events [pathological fractures and vertebral collapse, bone infarcts, osteonecrosis requiring hip replacement]) and decreased bone mineral density, along with neurological disorders in some rarer forms [3]. The age of diagnosis varies considerably (from birth to 81 years), though half of patients are under 10 years of age at the time of diagnosis [3]. The disease’s progression is also highly variable; some patients remain asymptomatic until their adult years, whereas others develop signs and complications during their early childhood [4,5]. GD is traditionally classified into three clinical types according to the presence or absence of neurological impairment [6]. The type 1 or chronic non-neuropathic form, defined by the absence of neurological findings, is the most frequent form of the disease (94% of patients) [3], affecting both children and adults [7]. Treatment of GD is based on the evaluation of signs of disease progression, mainly hematological (i.e., thrombocytopenia, anemia) and/or visceral (i.e., splenomegaly, hepatomegaly) and/or bone-related manifestations, described in the GD Diagnosis and Treatment national guidelines (PNDS) [8]. Serum chitotriosidase, tartrate-resistant acid phosphatase (TRAP), angiotensin-converting enzyme (ACE) and ferritinemia can be used to evaluate disease progression [5,9]. In 2009, two treatments granted Marketing Authorizations were available: enzyme replacement therapy (ERT) using imiglucerase (Cerezyme® [Alglucerase® before 1996], Genzyme, a SANOFI company) and substrate reduction therapy (SRT) by miglustat (Zavesca®, ACTELION). The reference treatment for GD is ERT. The recommended posology for imiglucerase in France at initiation of treatment is 60 U/kg/14 days [10], subsequently dosing can be individualized according to the patient’s achievement of stabilization of parameters, and the regression or normalization of signs and/or symptoms that initially led to treatment indication [8,10-17]. Once instituted, ERT is generally administered for life [10,18].

Temporary manufacturing difficulties encountered at Genzyme’s manufacturing plant led to a worldwide significantly constrained imiglucerase supply between June 2009 and November 2010. Treatment recommendations were implemented with the European Medicines Agency (EMA); high-risk groups were identified and prioritized to receive imiglucerase and included infants, children, adolescents and adult patients at high risk for the development of severe, life-threatening disease progression [19,20]. In France, joint recommendations were issued by the GD treatment evaluation committee (Comité d’Evaluation du Traitement de la maladie de Gaucher [CETG], the French Medicines Agency [L’Agence française de sécurité sanitaire du médicament et des produits de santé (Afssaps), currently L’Agence nationale de sécurité du médicament et des produits de santé (ANSM)], representatives of pharmaceutical companies, and representatives of patient associations (the Vaincre les Maladies Lysosomales [VML], and French representatives of the European Gaucher Alliance). Similarly, close clinical and biological monitoring of hematological parameters (hemoglobin and platelet counts) and biomarkers (chitotriosidase) was recommended during this period [21,22]. The management of the imiglucerase supply constraint in France proceeded in several steps: first, a recommendation of a 50% dose reduction for patients with moderate disease (June 2009), followed by a subsequent recommendation of a 50% dose reduction in children, adolescents, GD3 patients, and pregnant women, and a switch to miglustat or a discontinuation of imiglucerase for other patients (Aug 2009). In September 2010, a letter from the EMA (Direct Healthcare Professional Communication) informed health care professionals of an improvement in supply of imiglucerase such that patients who were being treated with reduced doses could be returned to doses per SmPC, but patients could not be initiated on imiglucerase or switched back to imiglucerase. Under the circumstances, two other ERTs in development at the time were rapidly made available to French treating physicians: velaglucerase alfa (VPriv®, SHIRE) and taliglucerase alfa (PROTALIX-PFIZER). Resumption of imiglucerase production in October 2010 allowed for treatment to be progressively resumed or doses to be adjusted to individual requirements.

The goal of this study was to evaluate the clinical and biological impact of the imiglucerase supply constraint in France from 01 June 2009 to 31 October 2010 (17 months).

## Methods

### Design and regulatory authorizations

This was an observational, retrospective, multicenter, national, non-comparative study.

All patients with GD1 or GD3, treated with imiglucerase (Cerezyme® [Ceredase® before 1996], Genzyme-a SANOFI company) for at least 6 months at the time of their therapeutic scheme modification, which was due to the imiglucerase supply constraint, could potentially be included in this study. To this aim, all French physicians treating at least one patient with GD at the time of the study implementation were proposed to participate as study investigators.

The patient (or legal guardian) was informed and agreed to participation. The study was approved by the French Healthcare Research Data Processing Advisory Board, the French National Data Protection Commission, and the Institutional Review Board of Paris North Hospitals. It was conducted in accordance with the French Good Epidemiological Practice guidelines.

### Data collected

The study comprised a single visit for each patient that took place between May 2011 (first patient) and April 2012 (last patient). During this visit, the following data were collected: demographic data; information about GD history (age at time of diagnosis, phenotype, genotype, prior history of splenectomy, bone impairments, and alglucerase/imiglucerase treatment history); imiglucerase treatment status; biological and clinical characteristics (notably bone manifestations) in the 6 months preceding the first treatment modification; subsequent changes in the imiglucerase treatment regimen (dose modification, discontinuation, switch to other GD treatments); and evolution of clinical (weight) and biological parameters (hemoglobin and platelet counts, chitotriosidase, TRAP, ferritinemia, ACE) during the course of the constraint period. GD-related events occurring after the first modification to the patient’s therapeutic scheme, i.e., clinical or biological events considered potentially related to GD (rather than treatment-related) were also spontaneously reported by investigators. Among those, bone events were of special interest; they were defined in the statistical analysis plan as symptomatic bone events (as per PNDS guidelines [8]) with a radiological confirmation: avascular necrosis (AVN) of an epiphysis, bone infarct, pathological fracture, or vertebral collapse. Other GD-related events could be spontaneously reported by each physician such as hematological events (e.g., platelets and/or hemoglobin decrease) and visceral events (e.g. liver and/or spleen volume increase).

### Definition of constraint period

The absolute supply constraint period for the population was defined as starting on 01 June 2009 and ending on 31 October 2010. The start for each patient was defined as the date of the first imiglucerase treatment modification on or following 01 June 2009. For each patient, the constraint period extended from the time of first treatment modification to 31 October 2010.

For each parameter, the pre-constraint value was defined as the most recent evaluation within 6 months prior to the start of treatment modification for the patient.

### Statistical methods

All statistical analyses were computed with SAS software (version 9.1; SAS Institute; Cary, North Carolina, United States). Categorical variables were summarized using frequencies and percentages. Continuous variables were summarized by the number of non-missing observations, mean, standard deviation, median, minimum, and maximum (range). Analyses were mainly descriptive and statistical tests were performed for exploratory purposes. A two-sided type I error rate of 0.05 was used to define statistical significance.

The primary population for all analyses was the full analysis set (FAS), which comprised all included patients who had experienced a treatment modification.

Since the study patients had various treatment modifications in the course of the *whole constraint period*, in order to isolate and assess the impact of imiglucerase treatment modification on the biological and clinical outcome of the patients, the analysis was performed on a constraint period limited to the period during which the treatment modification (discontinuation or reduced dose compared to pre-constraint dose) was stable for at least 3 consecutive months in a given patient (‘*limited constraint period’*). Two subgroups of patients were defined according to the first modification lasting at least 3 consecutive months:

- Subgroup 1: Discontinuation of imiglucerase without addition of another GD treatment (i.e., miglustat (Zavesca®, ACTELION) or another ERT).
- Subgroup 2: Treatment with a reduced imiglucerase dose (in U/kg/4-weeks) compared to pre-constraint dose. The changes in biological parameters over time during the constraint period were analysed using mixed-linear models for repeated-measures, with intercept and slope considered as random.

Analysis of GD-related events was performed on the FAS during the whole constraint period, and for each subgroup during the constraint period limited to stable modification. We described the number and nature of GD-related bone events before and after the supply constraint onset, i.e., during the whole study period (from the time of first treatment modification on or after 01 June 2009 to 31 October 2010) and during the preceding equivalent time period (i.e., approximately 18 months, from January 2008 to June 2009). Finally, time to occurrence and probability to present a bone, hematologic or visceral event during the limited constraint period was estimated with the Kaplan-Meier method.

## Results

### Patients’ characteristics

The characteristics of the 99 patients with GD 1 included in the study are described in Table 1. These patients aged from 7 to 84 years (median of 47 years); there were slightly more male patients (52.5%). Ten patients (10%) were under 18 years old. A splenectomy had been performed for 30% of the patients (partial for one patient). During the 18 months preceding the initial therapeutic scheme modification, we identified 16 bone events (5 AVN, 10 bone infarcts and 1 vertebral compression) in 12 patients. Evaluation within the 6 months preceding the initial therapeutic scheme modification (pre-constraint timepoint) showed splenomegaly for 36% of the 69 non-splenectomized patients. Hepatomegaly and asthenia were reported for 30 patients each (30%). Bone impairment was reported for 33% of the patients, consisting mainly of localized bone pain (55% of the cases) and osteoporosis (21%). No recent bone crisis or fractures were reported during this timeframe. Other clinical manifestations were reported for 15 patients (15%; mainly gingival bleeding), and concomitant diseases were reported for 38 patients (38%; mainly hypertension). The pre-constraint characteristics were mostly comparable for patients who discontinued imiglucerase and for patients who were treated with a reduced dose for at least 3 consecutive months, excepting median age because none of the pediatric patients discontinued imiglucerase.

**Table 1:** **Patients characteristics at baseline, overall and for two subgroups**

	Total (N = 99)*		**Patients who discontinued imiglucerase for at least 3 consecutive months (N = 61)**		**Patients receiving a reduced 4-week imiglucerase dose for at least 3 consecutive months (N = 32)**	
	**n**	**Median (range) or n (%) patients**	**n**	**Median (range) or n (%) patients**	**n**	**Median (range) or n (%) patients**
Gender	99		61		32	
Male		52 (52.5%)		32 (52.5%)		17 (53.1%)
Female		47 (47.5%)		29 (47.5%)		15 (46.9%)
Age (years)	99		61		32	
At inclusion		47 (7; 84)		47 (22 ; 84)		30 (7; 71)
At GD diagnosis		20 (1; 64)		23 (2; 61)		8.5 (1; 64)
Weight (kg)	85	66 (29; 105)	51	72 (45; 105)	28	60 (29; 92)
Genotyping performed	99	56 (56.6%)	61	35 (57.4%)	32	18 (56.3%)
If yes, mutations:	56		35		18	
L444P/Other		2 (3.6%)		2 (5.7%)		-
N370S/L444P		10 (17.9%)		10 (28.6%)		-
N370S/N370S		8 (14.3%)		5 (14.3%)		2 (11.1%)
N370S/Other		25 (44.6%)		10 (28.6%)		13 (72.2%)
Other/Other		11 (19.6%)		8 (22.9%)		3 (16.7%)
Treated with alglucerase before 1996	99	23 (23.2%)	61	13 (21.3%)	32	8 (25.0%)
Hepatomegaly	98	30 (30.6%)	60	18 (30.0%)	32	9 (28.1%)
Splenectomy	99	30 (30.3%)	61	21 (34.4%)	32	7 (21.9%)
Splenomegaly in non-splenectomized patients	69	25 (36.2%)	40	13 (32.5%)	25	10 (40.0%)
Asthenia	99	30 (30.3%)	61	20 (32.8%)	32	7 (21.9%)
Other clinical manifestation(s)	99	15 (15.2%)	61	7 (11.5%)	32	6 (18.8%)
Concomitant disease	99	38 (38.4%)	61	25 (41.0%)	32	11 (34.4%)
Bone impairment	99	33 (33.3%)	61	22 (36.1%)	32	8 (25.0%)
Hemoglobin (g/L)	85	140 (104; 163)	52	143 (106; 163)	27	134 (104; 154)
Platelets (10^3^/mm^3^)	86	182.5 (48; 478)	52	179.5 (48; 478)	28	185 (59; 415)
Ferritin (μg/L)	50	221 (16; 2310)	33	216 (16; 1150)	13	275 (29; 2310)
Chitotriosidase (nmol/mL/h)	64	730 (3; 16300)	38	234 (14; 16300)	20	1251 (3; 10170)
ACE (IU/L)	42	51.5 (10; 252)	26	42.5 (10; 252)	13	70 (19; 171)

*6 patients are not reported in the subgroups: 2 patients could not be classified (no stable modification for at least 3 consecutive months) and 4 patients took only miglustat for at least 3 consecutive months.

n: number of patients with available data.

### Treatment modifications

The first treatment modification (i.e., on or following 01 June 2009) took place at a mean time of 8 (±4) years after imiglucerase initiation. The doses of imiglucerase provided during the constraint period are presented in Table 2. Overall, the median dose of imiglucerase was 116 U/kg/4-weeks (range: 51; 267) at treatment initiation and 92 U/kg/4-weeks (range: 10; 239) prior to modification. At the first modification, the median dose of imiglucerase decreased to 40 U/kg/4-weeks, with a median absolute reduction of −52 U/kg/4-weeks (range: −127; −6). At 3, 6 and 9 months after the first modification, the dose of imiglucerase ranged from 0 (discontinuation) to a maximum of 170 U/kg/4-weeks, with a median absolute change of 69, −51 and −29 U/kg/4-weeks, respectively. Twelve months after the first modification, the imiglucerase dose ranged from 0 to 129 U/kg/4-weeks, with the median dose of 0 due to 55% of the patients having discontinued treatment with imiglucerase. Similar results were observed at the last modification prior to 01 November 2010, with 58% of patients having discontinued treatment with imiglucerase and doses ranging from 0 to 160 U/kg/4-weeks. When the patients who discontinued treatment (dose = 0) were excluded, the median dose of imiglucerase was 49 U/kg/4-weeks (range: 14; 181) at the first modification, progressively increasing overtime to 84 U/kg/4-weeks (range: 14; 160) at the last modification.

**Table 2:** **Evolution of imiglucerase dose during the supply constraint period**

		**Dose of imiglucerase (U/kg/4-weeks)**			**Change from pre-constraint dose (U/kg/4-weeks)**		
		**n**	**Mean (SD)**	**Median (range)**	**n**	**Mean (SD)**	**Median (range)**
Pre-constraint (in the 6 months prior to first modification)		94	92.5 (33.5)	92 (10; 239)			
First modification on or after 01 Jun 2009		99	35.0 (32.0)	40 (0; 181)	94	−55.9 (28.9)	−52 (−127; −6)
Time interval after the first modification:	3 months	98	17.5 (29.4)	0 (0; 160)	93	−74.1 (38.6)	−69 (−223; 0)
	6 months	95	41.0 (42.8)	49 (0; 160)	91	−51.1 (51.6)	−51 (−223; 62)
	9 months	95	55.9 (45.0)	56 (0; 170)	90	−36.6 (48.1)	−29 (−223; 62)
	12 months	96	37.3 (45.5)	0 (0; 129)	91	−55.8 (52.2)	−60 (−223; 62)
Last modification prior to 01 Nov 2010		98	37.6 (47.3)	0 (0; 160)	93	−56.2 (49.7)	−64 (−130; 62)

n: number of patients with available data; SD: standard deviation; Range: minimum; maximum.

**Figure 1:**
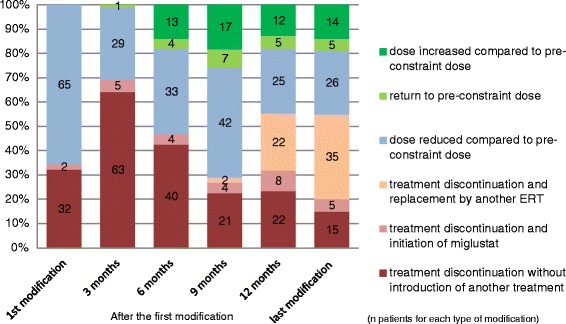
Number (bars) and percentage (y-axis) of patients for each type of modification of imiglucerase treatment during the supply constraint. ERT: enzyme replacement therapy.

For the overall population of GD 1 patients included in this study, the mean duration of modification was 15 (±2) months. During this period, treatment modifications reached a median number of 4, with a median average duration of approximately 4 months for each patient. The treatment was modified once for 7 patients (7%), twice for 16 patients (16%), between three and six times for 59 patients (60%) and more than six times for 17 patients (17%). The types of modification over time (i.e., for a given patient, from the date of first modification until 31 October 2010) are presented in Figure 1.

Discontinuation of imiglucerase treatment without introduction of another treatment was reported for 32 patients at the first modification and for 63 patients 3 months after the first modification. Discontinuation of imiglucerase treatment progressively decreased to 15 patients by the last modification prior to 1 November 2010. At this last time point, replacement of imiglucerase by another ERT was reported for 35 patients (velaglucerase for 25 patients and taliglucerase for 10 patients).

A dose reduction of 50% or more was reported for 30 patients (31%) at the first modification and 5 to 10 patients at subsequent timepoints. At 6 months after the first modification and at subsequent timepoints, 4% to 8% of the patients could return to the pre-constraint dose, and 14% to 18% of the patients were treated with higher doses compared to pre-constraint doses.

### Evaluation of the impact of imiglucerase treatment modifications on the disease

The progression of the biological parameters was assessed on a period limited to the duration of the first stable modification. This period lasted 3 to 15 months depending on each individual, with a median of 5 months in the subgroup of 61 patients who discontinued imiglucerase, and a median of 6 months in the subgroup of 32 patients treated with a reduced dose (6 patients could not be analysed in either of the subgroups as they had no such stable modification).

#### Biological parameters

The results of mixed linear models used to assess the development of biological parameters are presented in Table 3. These results must be analysed with caution due to the limited available data and variability in the time at which laboratory parameters were collected. Too few data were available for TRAP analysis and no quantitative measures of bone involvement were systematically evaluated.

**Table 3:** Description of the constraint period limited to duration of first stable modification*

	**Patients who discontinued imiglucerase (N = 61)**			**Patients receiving a reduced imiglucerase dose (N = 32)**		
**Imiglucerase treatment**						
	n	Median (range)		n	Median (range)	
Time between treatment start and first modification (years)		9.7 (1; 14)			8.4 (1; 13)	
Duration of the limited period of constraint (months)		5.1 (3; 15)			6.0 (3; 15)	
Imiglucerase dose (U/kg/4-weeks):						
At treatment initiation	58	118.5 (51; 246)		31	113.1 (58; 267)	
At pre-constraint timepoint	56	89.4 (10; 223)		32	94.7 (46; 239)	
At first modification	61	0.0 (0.0; 0.0)		32	52.4 (32; 181)	
*Dose decrease from pre-constraint dose (%):*					*n (%) patients*	
*<25%*					*8 (25.0%)*	
*≥25%; <50%*					*13 (40.6%)*	
*≥50%; <75%*					*11 (34.4%)*	
**Evolution of biological parameters**						
	n	Slope Estimate (SE)	Time-effect (p-value)	n	Slope Estimate (SE)	Time-effect (p-value)
Hemoglobin (g/L)	47	−0.8 (0.2)	**0.003**	26	0.6 (0.4)	0.196
Platelets (10^3^/mm)	47	−5905 (1721)	**0.002**	26	1762 (1720)	0.322
Serum ferritin (μg/L)	22	12.4 (6.4)	0.078	10	−3.4 (13.3)	0.812
Chitotriosidase (nmol/mL/h)	31	537 (208)	**0.021**	15	179 (259)	0.510
ACE (IU/L)	16	3.6 (1.1)	**0.012**	11	−2.8 (1.9)	0.228
**Spontaneously reported GD-related events (by MedDRA Preferred Term)**						
	Number (%) of patients			Number (%) of patients		
**All events**	15 (24.6%)			7 (21.9%)		
**Bone events**						
Bone infarction	1 (1.6%)			-		
Hand fracture	1 (1.6%)			-		
**Hematological events**						
Thrombocytopenia	3 (4.9%)			1 (3.1%)		
Pancytopenia	1 (1.6%)			-		
Epistaxis	1 (1.6%)			1 (3.1%)		
Gingival bleeding	1 (1.6%)			-		
Hematoma	1 (1.6%)			-		
**Visceral events**						
Hepatomegaly	-			1 (3.1%)		
**Other events**						
Asthenia/ Fatigue	4 (6.6%)			3 (9.4%)		
Bone pain	2 (3.3%)			1 (3.1%)		
Musculoskeletal pain	-			1 (3.1%)		
Arthralgia	1 (1.6%)			-		
Serum ferritin increased	-			2 (6.3%)		
ACE increased	-			1 (3.1%)		
Chitotriosidase increased	1 (1.6%)			-		
Parkinson’s disease	1 (1.6%)			-		
Stress	1 (1.6%)			-		

*”First stable modification” refers to discontinuation or reduced dose for at least 3 consecutive months.

Numerical values in bold print indicate statistical significance.

In the subgroup of 61 patients who discontinued imiglucerase, the models showed a statistically significant decrease in hemoglobin (p = 0.003) with a slope estimate of −0.8 g/L/month, and in platelets (p = 0.002) with a slope estimate of −5905.10^3^/mm^3^/month. There was a statistically significant increase in chitotriosidase (p = 0.021) and in ACE (p = 0.012), but only 16 patients had available data for ACE.

Changes over time observed in the models for hemoglobin, platelets, ferritin, chitotriosidase and ACE in the subgroup of 32 patients treated with a reduced imiglucerase dose were not statistically significant.

#### Spontaneously reported GD-related events

##### Events reported during the whole supply constraint period

From the first modification for a given patient until the end of the constraint period (31 October 2010), 64 GD-related events were spontaneously reported by the investigators for 39 patients (39%) (Table 4). The majority of these events (84%) were of mild or moderate intensity. Most events resolved (32 events, 50%) or improved (16 events, 25%), one event (1.6%) aggravated, and the remaining (15 events, 23.4%) persisted without change (terms reported by investigators were “ongoing,” “stable,” “persisting,” and “no change”). Among these GD-related events, 8 bone events were spontaneously reported by the investigators in 8 patients: bone infarction (four patients), and osteonecrosis, femur fracture, hand fracture and rib fracture (one patient each). All patients except one had previous history of bone impairment. Two events of severe intensity (bone infarction and rib fracture) led to a significant deterioration of the patient’s health, according to the physician.

**Table 4:** GD-related events spontaneously reported during the whole period of imiglucerase supply constraint*

	**GD Type 1 (N = 99)**	
	**Number of events**	**Number (%) of patients with at least 1 GD-related event**
**All events**	**64**	**39 (39.4%)**
**Bone events**	**8**	**8 (8.1%)**
Bone infarction	4	4 (4.0%)
Osteonecrosis	1	1 (1.0%)
Femur fracture	1	1 (1.0%)
Hand fracture	1	1 (1.0%)
Rib fracture	1	1 (1.0%)
**Other spontaneously reported events**		
***Hematological events***	***12***	***11 (11.1%)***
Thrombocytopenia	5	5 (5.1%)
Pancytopenia	1	1 (1.0%)
Epistaxis	3	3 (3.0%)
Gingival bleeding	1	1 (1.0%)
Platelet count decreased	1	1 (1.0%)
Haematoma	1	1 (1.0%)
***Visceral events***	***4***	***3 (3.0%)***
Hepatomegaly	2	2 (2.0%)
Hepatosplenomegaly	1	1 (1.0%)
Splenomegaly	1	1 (1.0%)
Other frequent events (incidence ≥3%)*	***24***	***19 (19.2%)***
Asthenia/Fatigue	8	8 (8.1%)
Serum ferritin increased	5	5 (5.1%)
Chitotriosidase increased	4	4 (4.0%)
Bone pain	4	4 (4.0%)
Osteopenia	3	3 (3.0%)

*Whole period of imiglucerase constraint refers to the period of time from first modification due to constraint to 31 October 2010).

Events are presented by MedDRA Preferred Term.

It should be noted that several events could be reported in one patient. During the considered period, a total of 64 events were reported in 39 patients: one event was reported in 26 patients, two events were reported in 5 patients, three events were reported in 4 patients and four events were reported in 4 patients.

*Sixteen other events (incidence <3%) were reported in a total of 12 patients (12.1%). These events were: tendonitis, angiotensin converted enzyme increased (2 patients each), arthralgia, back pain, coccydynia, musculoskeletal pain, thrombocytosis, increased blood acid phosphatase, Parkinson’s disease, sciatica, tremor, spontaneous abortion, stress, erectile dysfunction (1 patient each).

Three patients reported visceral events (hepatomegaly with or without splenomegaly), but none were of severe intensity.

Apart from these events of special interest, the most frequently reported GD-related event was asthenia/fatigue (8%). Some biological changes were also spontaneously reported by the investigators, the most frequent being thrombocytopenia, increased serum ferritin (each reported in 5% of the whole study population) and increased chitotriosidase (4%). Overall, hematological events were reported for 11 patients (11%).

*GD-related events reported during the limited constraint period: comparison between the subgroup of patients who discontinued imiglucerase and the subgroup of patients treated with a reduced dose of imiglucerase.*

As shown in Table 3, the percentage of patients who experienced at least one GD-related event during the constraint period that was limited to the duration of the first stable modification (median time 5.1 months for patients who discontinued and 6 months for patients with a reduced dose) was similar for the two subgroups of patients who discontinued imiglucerase and patients treated with a reduced dose (24.6% and 21.9%, respectively) of imiglucerase. No bone, hematological or visceral event led to significant deterioration of health in either subgroup, although the median duration of this period for the analysis was not long. Except for 3 outliers with clinically significant weight gain, weight remained mostly stable over time in both subgroups.

**Figure 2:**
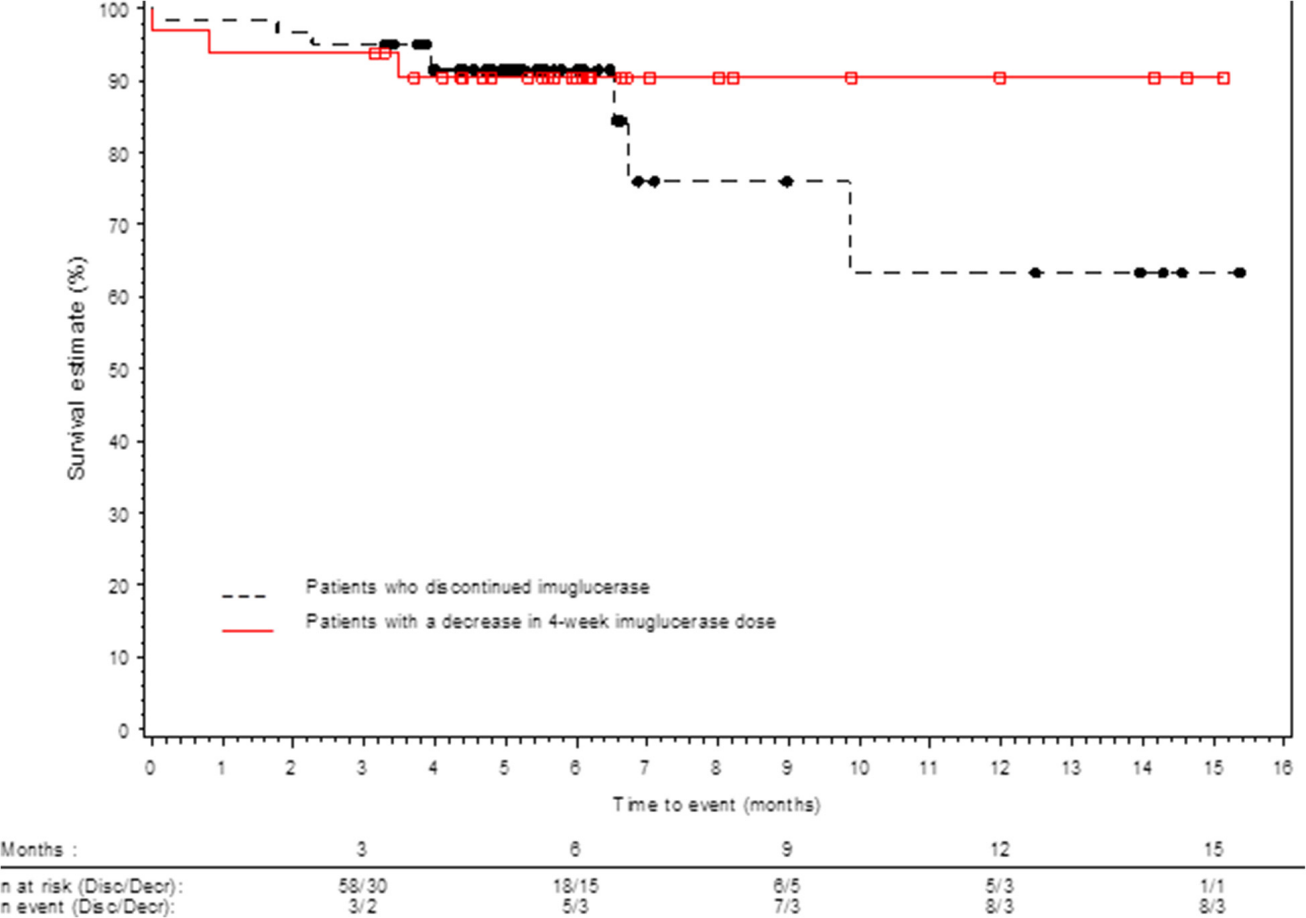
Kaplan Meier curve of time to first occurrence of a bone, hematologic or visceral event.

According to Kaplan-Meier estimation, the probability that a patient would present with a bone, hematological, or visceral event during the constraint was 23%. This probability was 37% for patients who discontinued the treatment for at least 3 consecutive months and 10% for patients treated with a reduced dose compared to the pre-constraint dose for at least 3 months (Figure 2).

## Discussion

This observational, retrospective, multicenter, national study evaluated the effects of the imiglucerase supply constraint in France between June 2009 and November 2010. According to the data from the French GD registry, 208 GD patients were treated with imiglucerase in June 2009 [23]. The number of included patients (99 patients with GD1) represents half of the potential number of eligible patients based on the French GD registry data. This difference might be explained by the fact that both physicians and patients may have been reluctant to participate in this observational study during the constraint period. Therefore, a selection bias in the population of this study cannot be excluded. Following the EMA and CETG recommendations, treatment was maintained for patients with the most at risk disease forms and these patients were less subject to dose reductions. The characteristics of the patients included in the present study were generally comparable to those reported for a larger cohort in the French GD Registry [23], with slightly better results for biological parameters.

In accordance with CETG recommendations on the therapeutic management of pediatric patients during the constraint period, none of the 10 patients under 18 years old discontinued imiglucerase and all were treated with a reduced dose of imiglucerase (17% to 58% dose reduction). The first treatment modification consisted of imiglucerase discontinuation for 34% of the whole study population, and a reduced imiglucerase dose without the introduction of another treatment for 66%. The dose reduction was over 50% compared to the pre-constrained dose for nearly half of these patients. Progressive re-supplying reduced the number of patients who had discontinued imiglucerase from 70% to 29% between 3 and 9 months after the first modification. A return to pre-constraint doses, or even higher doses, was possible for 18% to 26% of the patients 6 to 12 months after the first modification. However, the available imiglucerase supply decreased again in April 2010 and, at the last treatment modification prior to 01 November 2010, 58% of patients had discontinued imiglucerase treatment, including 37% who started treatment with another ERT, and 5% who started miglustat. These results were consistent with the treatment recommendations issued in September 2010 by the EMA, which dictated a progressive return to normal dose for patients who were treated with reduced doses, but no new initiation of treatment with imiglucerase or switch back to imiglucerase for patients who had discontinued.

It was anticipated that the impact of the imiglucerase supply constraint on French Gaucher disease patients would be difficult to interpret overall because of the variability in local treatment management and the numerous consecutive dose changes according to supply availability. As a result, two subgroups of patients were defined for this analysis: a subgroup of 61 patients who discontinued imiglucerase, and a subgroup of 32 patients who were treated with a reduced imiglucerase dose for a period lasting a minimum of 3 consecutive months, without addition of another treatment for Gaucher disease. Results for these subgroups were analysed for the period of constraint limited to stable imiglucerase treatment regimen, relative to each patient. Six patients were not reported in the subgroups: 2 patients could not be classified (no stable modification for at least 3 consecutive months) and 4 patients took only miglustat for at least 3 consecutive months.

This study has numerous methodological shortcomings that limit the extent of interpretation, e.g., retrospective data collection, an imbalance in statistical power between the two compared subgroups of patients due to disproportionate sample size, a small number of available data for some of the biological parameters studied, and a limited robustness of statistical models due to variability in the time of retrospective data collection. One should also mention that systematic quantitative bone and visceral follow-up measures were not available, which limited the outcome evaluation in the study population.

Taking into account these limitations, the study results indicate that treatment discontinuation has a greater impact than imiglucerase dose reduction on the progression of biological parameters. Indeed, a statistically significant decrease in hemoglobin and platelet counts and an increase in chitotriosidase and ACE were observed in the subgroup of patients who discontinued the treatment for at least 3 consecutive months.

The results observed in this French study are consistent with those reported on several smaller cohorts in different countries. The impact of the supply constraint has previously been described on 26 patients from Israel who discontinued treatment with imiglucerase [24], 34 Italian patients with an imiglucerase dose reduction for at least 1 year [25], 26 patients from Netherlands and United Kingdom who switched from imiglucerase to velaglucerase [26], 50 Spanish patients who discontinued imiglucerase treatment or were treated with a reduced dose (50% reduction for most) analysed during a 6-month period [27], 24 Australian patients put on a “drug holiday” during the supply constraint [28], and 14 patients from Taiwan who had their imiglucerase dose reduced from 60 to 30 U/kg/2-weeks [29]. The cohorts’ characteristics were mostly comparable to the French cohort, but the imiglucerase doses before the constraint were generally lower (median of 30 to 60 U/kg/4-weeks, except for Taiwan with a median of 120 U/kg/4-weeks). All these studies reported a significant increase in chitotriosidase and slight decreases in hemoglobin and platelets in patients who either discontinued the treatment or had a pronounced dose reduction.

During the whole period analysed in this study, hematological, visceral and bone events were spontaneously reported as GD-related events in 11%, 3% and 8% of patients, respectively. It must be noted that, among the 8 patients who experienced bone events during the constraint period, all but one had reported a previous history of bone events, and 50% had undergone total splenectomy. Splenectomy and a history of bone events before treatment initiation have been shown to be risk factors for developing bone events, even under treatment with ERT [23]. The rate of bone events observed during the whole supply constraint period (8%) was not superior to the rate observed during the 18-months pre-constraint period (12%). However, the data reporting was different between the two periods (systematic vs. spontaneous reporting), therefore any comparison should be made with caution. In addition, one should have in mind that GD is a chronic disease with a very high inertia; therefore, any impact (whether positive under treatment or negative after treatment interruption) on the visceral and bone compartments may take months or even years before it can be translated into clinically observable changes.

Furthermore, GD-related events relying on imaging data must be analysed with caution (e.g., spleen and liver volumes, bone lesions, bone infiltration, bone mineral density), because these data were not collected systematically in participating centers and the assessments were likely heterogeneous across centers because of technical differences (e.g., machines, protocol, reader). Similarly, it is unlikely that bone pain, whether localized or diffuse, was homogenously assessed across centers. In addition to limiting the extent to which clinical outcome can be evaluated in this study, these observations highlight a need for improvement regarding the systematic assessment of splenic and hepatic volumes, and of bone lesion progression using standardized magnetic resonance imaging.

As for the limited (to a stable modification) constraint period analysis, the percentage of patients who experienced at least one GD-related event was similar for patients who discontinued imiglucerase and those treated with a reduced dose (25% and 22%, respectively). However, according to Kaplan-Meier estimation, the probability for a patient to present a bone, hematological or visceral event during the limited constraint period was higher for patients who discontinued the treatment compared to patients who were treated with a reduced dose: 37% and 10%, respectively. According to recommendations, patients who discontinued the treatment were the ones with lesser disease burden, so an increase in GD-related events in this population compared to patients treated with a reduced dose could be linked to treatment discontinuation.

Taken altogether, these elements suggest that, in the context of a limited supply, it is preferable to maintain patients under treatment with reduced doses (or less frequent infusions) than to discontinue treatment with imiglucerase. These results are in line with the studies published before the supply constraint took place, which suggested that disease progression resumes following ERT interruption, and recommended avoiding this practice [30-33].

This study concerned a limited period of observation, hence could not allow the evaluation of the long-term impact of dose reduction. Some events, particularly bone events, may arise even with treatment continuation [34] or years after treatment discontinuation or reduction of treatment.

These observations prove useful to depict how the health care community can respond to such circumstances. The convening of expert meetings at the European (EMA) and French level (National Committee for GD Treatment Evaluation in coordination with the National Medicine Agency), as well as with patients’ associations and representatives of pharmaceutical companies allowed a timely issuing of treatment recommendations that, coupled with close case-by-case management at each physician’s level, limited the impact of the constraint on French Gaucher patients. The situation is now back to normal in France, allowing patients to return to their recommended imiglucerase dose with proven efficacy, as mentioned in the product Summary of Product Characteristics for imiglucerase [10]. In the context of full supply, appropriate individualized dosing is indeed necessary to achieve and maintain long-term therapeutic goals, specifically skeletal goals [12,13,15-18].

## Conclusions

The imiglucerase supply constraint in France between June 2009 and November 2010 involved a large inter-individual variability in the therapeutic management of patients with GD, with numerous treatment modifications depending on supply availability. Overall, the joint recommendations issued by the French Medicine Agency, the National Committee for GD Treatment Evaluation, patient associations, and pharmaceutical companies allowed for satisfactory management of GD patients during the supply constraint in France while protecting the most vulnerable patients. Keeping in mind the methodological limitations of this observational retrospective study without long-term follow-up, imiglucerase dose decreases have shown a lower impact on the clinical and biological outcomes in this population compared to treatment discontinuation. However, general effects, such as fatigue and bone pain, reported in patients treated at a reduced dose or with interrupted imiglucerase therapy emphasize the importance of maintaining appropriate individualized doses for each patient.

## Abbreviations

ACE: Angiotensin-converting enzyme
AVN: Avascular Necrosis
CETG: Comité d’evaluation du Traitement de la maladie de Gaucher (France)
EMA: European medicines agency
ERT: Enzyme replacement therapy
FAS: Full analysis set
GD: Gaucher disease
PNDS: Protocole national de diagnostic et de soins (Diagnosis and Treatment national guidelines)
SmPC: Summary of product characteristics
SRT: Substrate reduction therapy
TRAP: Tartrate-resistant acid phosphatase
